# Coffee consumption and the risk of cerebrovascular disease: a meta-analysis of prospective cohort studies

**DOI:** 10.1186/s12883-021-02411-5

**Published:** 2021-10-02

**Authors:** Lung Chan, Chien-Tai Hong, Chyi-Huey Bai

**Affiliations:** 1 Department of Neurology, Shuang-Ho Hospital, Taipei Medical University, No. 291, Zhongzheng Rd, Zhonghe District, New Taipei City, 23561 Taiwan; 2 Department of Neurology, School of Medicine, College of Medicine, Taipei Medical University, Taipei, Taiwan; 3 School of Public Health, College of Public Health and Nutrition, Taipei Medical University, 5/F Health Science Building, 250 Wu-Hsing Street, Taipei City, Taiwan; 4 Department of Public Health, College of Medicine, Taipei Medical University, Taipei, Taiwan

**Keywords:** Cerebrovascular disease, Coffee, meta-analysis, Cohort, Stroke

## Abstract

**Background:**

Stroke is a crucial health threat to adults worldwide. Despite extensive knowledge of risk-factor mitigation, no primary prevention exists for healthy people. Coffee is a widely consumed beverage globally. Health benefit of coffee for several neurological diseases has been identified; however, the association between stroke risk and coffee consumption in healthy people has not been determined. We investigated the effect of coffee on stroke risk by conducting a meta-analysis of prospective cohort studies.

**Methods:**

Electronic databases, namely PubMed, BioMed Central, Medline, and Google Scholar, were searched using terms related to stroke and coffee. Articles that described clear diagnostic criteria for stroke and details on coffee consumption were included. The reference lists of relevant articles were reviewed to identify eligible studies not shortlisted using these terms. Enrolled studies were grouped into three outcome categories: overall stroke, hemorrhagic stroke, and ischemic stroke.

**Results:**

Seven studies were included and all of them were large-scale, long-term, follow-up cohort studies of a healthy population. Upon comparing the least-coffee-consuming groups from each study, the meta-analysis revealed a reduction in the risk of overall stroke during follow-up (hazard ratio [HR] for overall stroke = 0.922, 95% confidence interval [CI] = 0.855–0.994, *P* = 0.035). In studies with a clear definition of hemorrhagic and ischemic stroke, coffee consumption reduced the risk of ischemic stroke more robustly than that of hemorrhagic stroke (hemorrhagic, HR = 0.895, 95% CI = 0.824–0.972, *P* = .008; ischemic, HR = 0.834, 95% CI = 0.739–0.876, *P* < .001). No obvious dose-dependent or U-shaped effect was observed.

**Conclusions:**

Coffee consumption reduces the risk of overall stroke, especially ischemic stroke. Further investigation is required to identify beneficial components in coffee, including caffeine and phenolic acids, to develop preventive medication for stroke.

**Supplementary Information:**

The online version contains supplementary material available at 10.1186/s12883-021-02411-5.

## Introduction

Stroke is a leading cause of mortality globally and results in more disabilities compared with other diseases [1]. In general, stroke can be separated into two subtypes: ischemic, which accounts for 80% of strokes, and hemorrhagic. The proportions of the two subtypes vary in different countries, which is associated with the national income [2]. The nonmodifiable risk factors of stroke include age and male sex, whereas the modifiable risk factors include hypertension, diabetes mellitus, hyperlipidemia, cardiovascular diseases, sedentary lifestyle, atrial fibrillation, smoking, and alcohol consumption [3]. Despite improved knowledge and control of modifiable risk factors, the decline in stroke incidence in the twenty-first century is unsatisfactory [4]. Acute treatments for stroke, especially ischemic stroke, have been developed, which have improved stroke recovery remarkably [5]. However, studies for identifying protective factors that can reduce the risk of stroke are warranted [6].

Coffee is a widely popular beverage worldwide. In addition to caffeine, coffee contains other biochemical compounds that affect human health [7]. Mounting evidence suggests noteworthy health benefits of coffee. Caffeine and phenolic compounds have antioxidant properties and regulate intracellular signaling for growth, proliferation, and apoptosis [8]; they also modulate the gut microbiome [9] and glucose and fat metabolism [10, 11]. The health benefits of coffee have been reported for diseases including cancer, cardiovascular disease, and Parkinson’s disease [12].

**Fig. 1:**
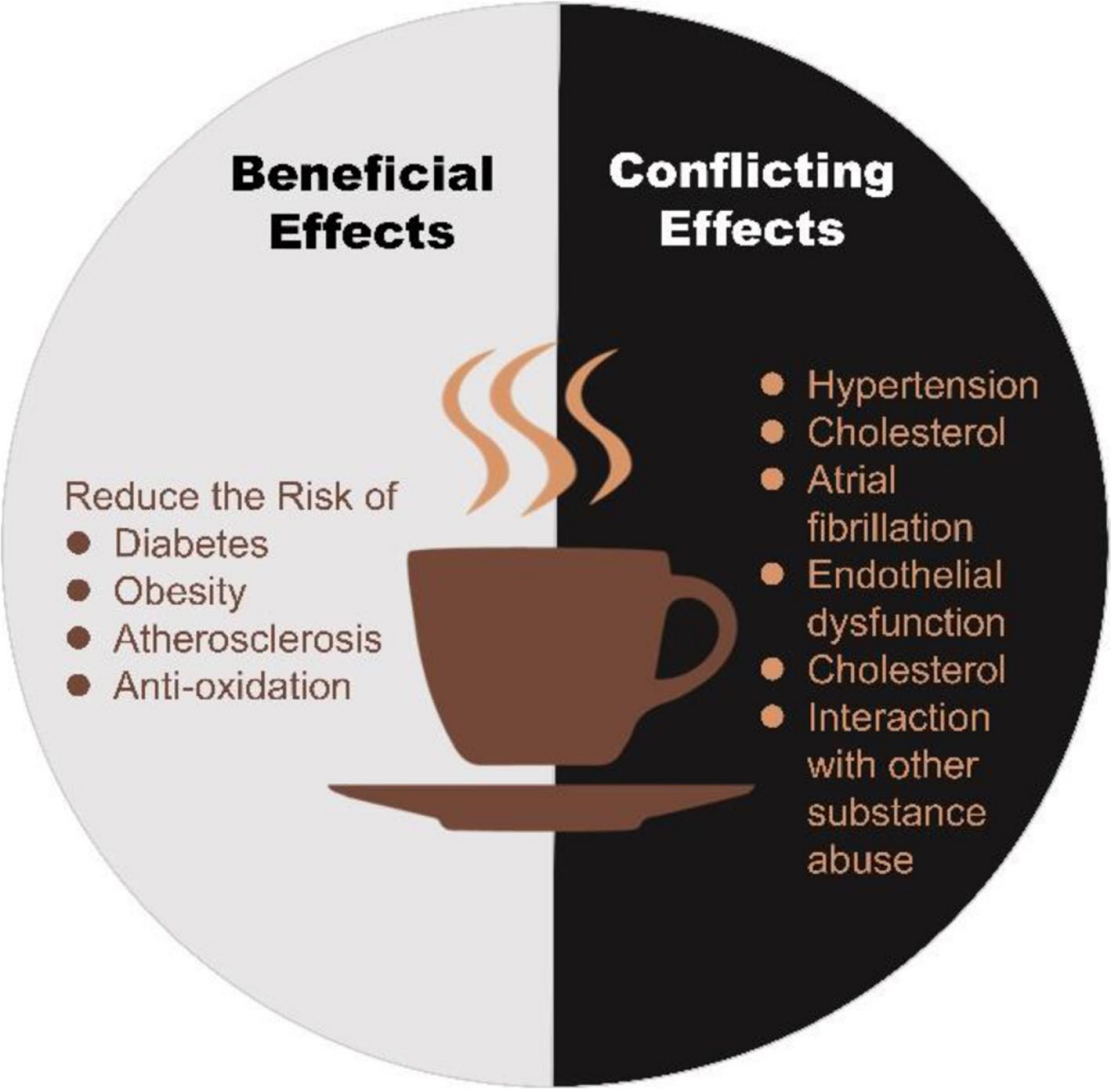
Relationship between coffee consumption and risk factors for stroke

The effect of coffee on stroke is unclear, and a U-shaped association has been claimed in some studies, which have suggested that the protective effect is dose dependent and a high dose of coffee is less effective [13, 14]. However, some studies have found consistent protection against stroke in moderate to heavy coffee drinkers [15, 16]. The mixed components and varied types of coffee may explain the discrepancy. A positive indication of coffee’s protective effects is that moderate coffee intake is associated with less carotid atherosclerosis in women who drink coffee occasionally [17]. For men, coffee intake is associated with reduced body weight [18]. Additionally, coffee consumption reduces the risk of diabetes [19]. Conversely, coffee may exacerbate certain risk factors for stroke. Caffeine, a major component of coffee, causes vasoconstriction and increases blood pressure. Habitual coffee drinking is associated with uncontrolled hypertension in older adults with hypertension [20], although a meta-analysis of other cohorts found no association between habitual coffee consumption and hypertension [21]. In addition, the sympathomimetic effect of coffee, especially caffeine, may be responsible for an increased risk of atrial fibrillation [22]. Unfiltered coffee may increase blood cholesterol, especially the undesirable low-density lipoprotein [23]. Moreover, confounding factors, including smoking, alcohol consumption, diet, education, and physical activity, interact with coffee regarding the risk of stroke [24]. Figure 1 summarizes the possible advantages and disadvantages of coffee regarding the risk of stroke. Because of the conflicting evidence, forming a reliable opinion regarding whether coffee consumption can reduce stroke risk and what amount of coffee provides the best protection is still challenging.

This study investigated the association between stroke risk and coffee consumption. To examine the role of coffee in primary stroke prevention, prospective cohort studies that enrolled healthy or stroke-free people with a record of coffee consumption were included in the meta-analysis. In addition to overall stroke, hemorrhagic stroke and ischemic stroke were subjected to subgroup analyses for risk assessment.

## Methods

### Literature search strategy

All relevant articles in English published from January 1, 1990, to May 31, 2020, were identified by searching PubMed, BioMed Central, Medline, and Google Scholar. Details regarding search terms are provided in the supplementary data. Moreover, the reference lists of relevant articles were reviewed to identify eligible studies not derived using the search terms. The meta-analysis procedure complied with PRISMA guidelines.

### Inclusion and exclusion of studies

**Fig. 2:**
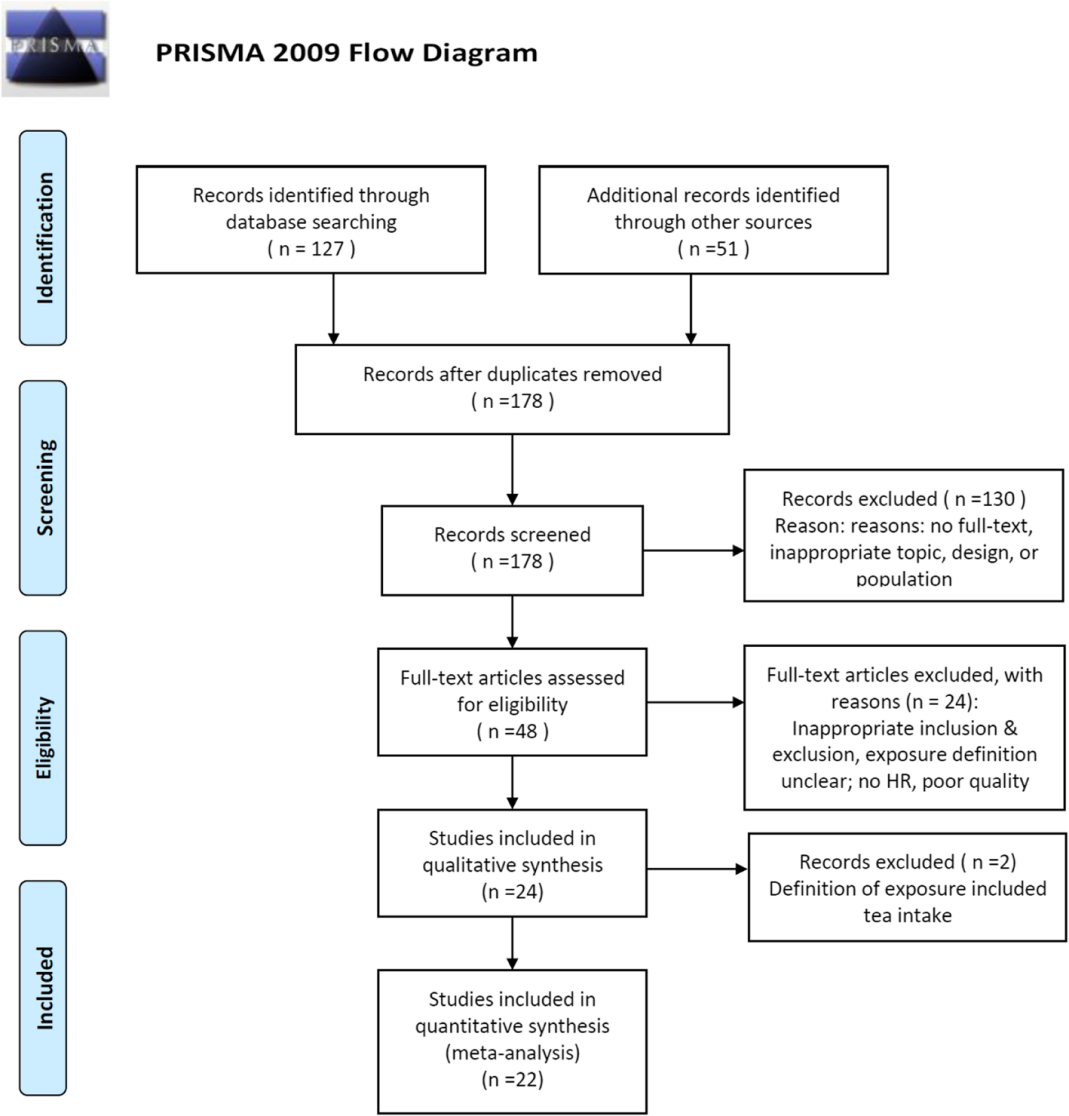
schematic of the literature search

The inclusion criteria were as follows: (1) clear definition of stroke diagnosis, (2) clear definition of the quantity of coffee consumed, (3) cohort study published as an original article, case series, or letter to the editor, and (4) publication in English. After ineligible studies were excluded, seven studies were ultimately included. The enrolled studies were grouped into three categories: those assessing overall risk of stroke (*n* = 7), those assessing ischemic stroke (*n* = 3), and those assessing hemorrhagic stroke (n = 3). The flow of the selection process is illustrated in Fig. 2.

### Data extraction

The following data were extracted: name of the first author, year of publication, country and location, study design, and diagnostic criteria for stroke. Three investigators (CH Bai, CT Hong, and L Chan) independently reviewed all data, and conflicts were resolved through consensus. Two investigators (CH Bai and YC Fan) independently extracted data from the seven candidate studies.

### Statistical analysis

Hazard ratios (HR) were determined, and 95% confidence intervals (CI) were calculated based on a binomial assumption. Furthermore, the I^2^ statistic was used to assess heterogeneity across the studies. All statistical analyses were performed using SAS (version 9.3, Statistical Analysis System, SAS.com, USA). All reported probability (*p*) values were two sided, with *P* < .05 considered statistically significant.

## Results

Of the seven studies (Table 1), four were conducted in Europe, one in the United States, and two in Japan [20, 25–30]. All seven studies enrolled healthy individuals without a history of stroke, and two studies included only female participants. All studies were large-scale, long-term, epidemiological cohort studies. Coffee consumption was evaluated using either detailed and comprehensive or simple questionnaires. Overall stroke, ischemic stroke, and hemorrhagic stroke were identified through self-reports, confirmation of medical records, or a national health care database. Most studies categorized caffeine consumption as degrees 3 to 5 points based on the number of cups of coffee per day or week, and only two studies provided *yes* or *no* options for regular coffee consumption.

**Table 1:** List of enrolled studies

Author	Country	Population	Follow-up (y)	Degree of coffee consumption (highest to lowest)	Outcome	Identification of stroke
Kokubo et al.	Japan	General population	13	5 (> 4 cups/d to 1–2 cups/wk)	Overall stroke, hemorrhagic stroke, ischemic stroke	Medical records
Floegel et al.	Germany	General population	8.9	4 (> 4 to 1 cup/d)	Overall stroke	Participant reports
Lopez-Garcia et al.	UK	Female nurses, women	5.0	4 (> 4 or less than 4 cups/wk)	Overall stroke, hemorrhagic stroke, ischemic stroke	Participant reports and medical records
Larsson et al.	Sweden	Women	10.4	3 (> 5 to 1–2 cups/d)	Overall stroke, hemorrhagic stroke, ischemic stroke	Medical registry
de Koning Gans et al.	Holland	General population	13	5 (> 6 to less than 2 cups/d)	Overall stroke	Medical registry
Mineharu et al.	Japan	General population	13.1	3 (> 3 to less than 1 cup/d)	Overall stroke	Medical Registry
Greenberg et al.	US	Elderly population	10.1	1 (> 1 cup/d)	Overall stroke	Participant reports

**Fig. 3:**
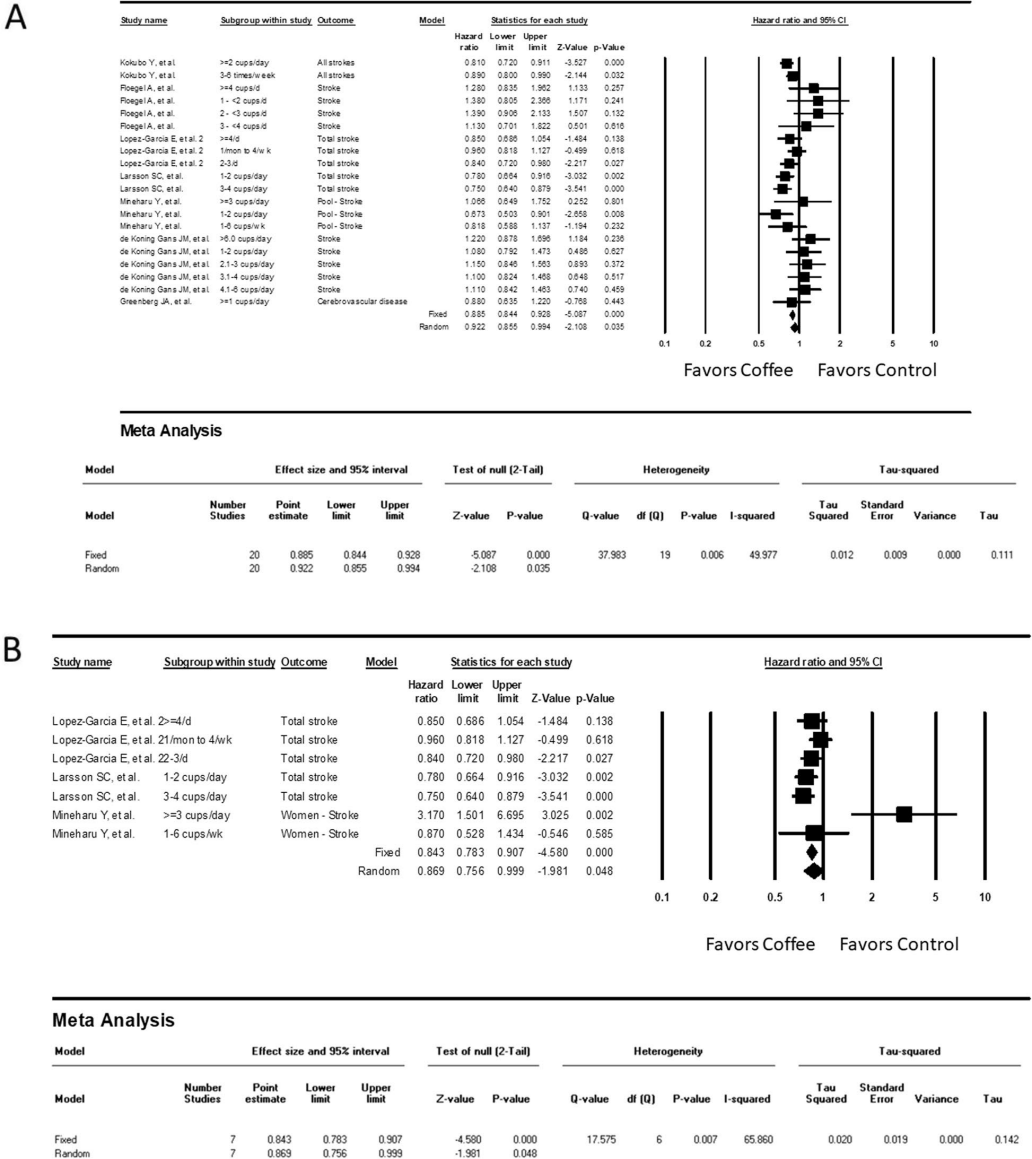
Forest plot of the hazard ratios (HRs) of overall stroke for (**A**) all participants and (**B**) female participants in the cohort studies

Considering variations in coffee consumption among studies, we considered results from all degrees of coffee consumers and considered the no-exposure group a reference to determine the HRs. Overall, 20 results extracted from the 7 studies were analyzed. Coffee consumption was significantly associated with a lower overall stroke risk (HR = 0.922, 95% CI: 0.855–0.994, *P* = .035 using the random model, I^2^ = 49.98%, Fig. 3A). Because two studies recruited only female participants and one study provided sex-segregated results, we further analyzed the association between coffee and stroke in female participants; coffee demonstrated a similarly considerable risk-lowering effect (HR = 0.869, 95% CI: 0.76–0.99, *P* = 0.048 using the random model, I^2^ = 65.86%, Fig. 3B).

**Fig. 4:**
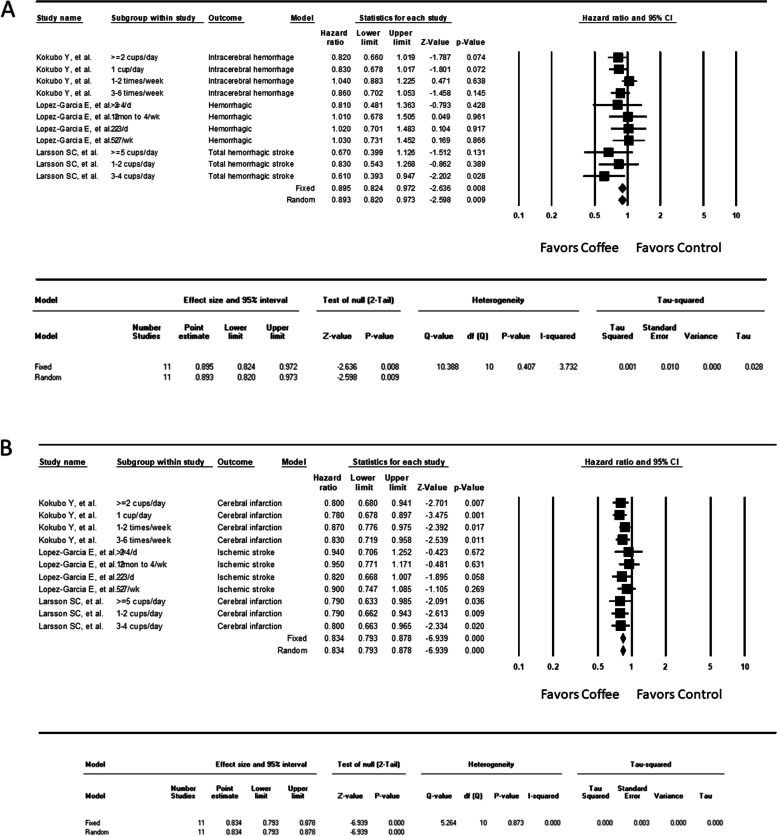
Forest plot of the hazard ratios (HRs) of (**A**) hemorrhagic stroke and (**B**) ischemic stroke among the participants in the cohort studies

Subgroup analyses were conducted to investigate the risk of ischemic and hemorrhagic stroke in coffee drinkers. Coffee consumption reduced the risk of ischemic more robustly than for hemorrhagic stroke (hemorrhagic, HR = 0.895, 95% CI = 0.824–0.972, *P* = .008; ischemic, HR = 0.834, 95% CI = 0.739–0.876, *P* < .001; Fig. 4A and B). Heterogeneity was unremarkable for both hemorrhagic and ischemic strokes (I^2^ = 3.732% for hemorrhagic and 0% for ischemic). No obvious dose-dependent or U-shaped effect (highest protection with moderate amounts of coffee and no protection with the highest amount) was observed under either condition.

## Discussion

Our study reveals the protective effect of coffee consumption against stroke, especially the overall stroke events and the risk of both ischemic and hemorrhagic stroke. Considering that most of the articles analyzed were large-scale, population-based cohort studies, this evidence indicates the benefit of coffee in primary stroke prevention.

Studies have reported the benefits of coffee in several health conditions [12]. Regarding neurological disorders, a meta-analysis conducted by Santos et al. demonstrated a protective effect of coffee on Alzheimer’s disease from pooled cohort studies, with a relative risk of 0.93 [31]. Compared with Alzheimer’s disease, the benefit of coffee on Parkinson’s disease is more substantial not only for reduced risk in healthy people but also for slowing disease progression in patients [32]. Additionally, coffee consumption reduces the risk of depression by 9% in elderly people who drink ≥4 cups of coffee per day [33] and reduces the risk of suicide by approximately one quarter for each increment of 2 cups/day of coffee according to three large-scale cohort studies of medical professionals [34].

Coffee also has beneficial effects on cardiovascular disease (CVD), another major vascular occlusive disorder. CVD shares several modifiable risk factors with stroke, such as hypertension, diabetes, hyperlipidemia, smoking, and obesity. On the disease- mechanism level, oxidative stress, inflammation, and endothelial dysfunction contribute to both diseases. The effect of coffee consumption reducing the risk of CVD has been established based on several large-scale cohort studies and pooled meta-analysis. A U-shaped protective effect, wherein the best protection is obtained for people who drink 3–5 cups of coffee per day, is presented as well. However, some distinctions exist between the two related diseases of CVD and stroke. First, CVD involves only ischemic infarction, whereas stroke results from either ischemic or hemorrhagic infarction. Second, cardioembolism, usually secondary to atrial fibrillation, causes stroke but not CVD. Because of these differences, the evidence for CVD cannot be applied directly to stroke. In the present meta-analysis, our findings reveal that for ischemic stroke, the net effect of coffee consumption is beneficial, with a considerable risk reduction of nearly 20%. We did not observe a U-shaped trend of risk reduction. The U-shaped phenomenon may not result solely from the effect of coffee. Biases may be introduced by socioeconomic status, use of other substances (e.g., cigarettes and alcohol) [35], and mental stress from high coffee consumption. The benefit of stroke risk reduction in people who consume coffee at different frequencies provides medical professionals an accessible and transparent approach to promoting coffee consumption for stroke prevention.

We detected heterogeneity within the studies included in the meta-analysis. Regarding overall stroke, Floegel et al. [26] reported a trend of increased stroke risk in their study on coffee consumers. The study was conducted in Europe with a mean follow-up of 8.9 years, and the authors reported that coffee consumption did not substantially increase the risk of chronic diseases (stroke, myocardial infarction, and cancer) but did protect against diabetes. Zhang et al. [36] reported a slightly increased stroke risk among women with diabetes. Thus, these studies have found that coffee consumption is associated with a decreased risk of CVD and all-cause mortality as well as better control of blood glucose. For other studies, a considerable trend in stroke protection has been observed.

Our findings reveal the stroke-preventive benefit of coffee consumption through a meta-analysis that included prospective, large-scale cohort studies with healthy or stroke-free individuals, and the follow-up spanned decades. The results are representative and support the benefit on coffee consumers. However, original epidemiological studies have not discussed the limitations. For instance, the accuracy of information regarding the frequency and amount of coffee consumption was questionable, although self-reported questionnaires are proven to be accurate and reproducible [37]. Another limitation is the lack of knowledge regarding the component responsible for the beneficial effect of coffee. Caffeine is postulated to be a key component, but the lack of similar benefits observed from tea and other caffeinated foods or beverages is confounding. Information on the type of coffee participants consumed was lacking. Lastly, none of the studies provided information on the subtypes of ischemic stroke, such as large-artery occlusion, cardioembolism, small-vessel diseases, or cryptogenic diseases, or whether coffee is beneficial for specific subtypes of ischemic stroke remains unknown.

## Conclusion

Our meta-analysis revealed an association between decreased stroke risk and coffee consumption; ischemic stroke was found to be the most preventable subtype of stroke. Coffee may be effective in primary stroke prevention, although the U-shaped relationship is a concern. Medical staff are encouraged to inform the population regarding coffee’s beneficial effects. Further analysis is warranted to identify the primary component of coffee responsible for preventing stroke.

## Supplementary Information

**Additional file 1.**

## Data Availability

Please contact the authors of the original studies included in the meta-analysis.
