# Supplementary material for: Coffee consumption and the risk of cerebrovascular disease: a meta-analysis of prospective cohort studies
Source: BMC Neurol. 2021 Oct 2;21:380. doi: 10.1186/s12883-021-02411-5 (PMC8487108; doi:10.1186/s12883-021-02411-5)
Supplement: Supplementary file 1 — Additional file 1. [file 12883_2021_2411_MOESM1_ESM.docx]

Supplementary information

1. Search keywords and strategy

((Cardiovascular disease[Title/Abstract]) OR (Cardiovascular diseases[Title/Abstract]) OR (cardiovascular diseases[Title/Abstract]) OR (cardiovascular disease [Title/Abstract]) OR (CVD[Title/Abstract]) OR (CVDs[Title/Abstract])) AND ((Caffeine[Title/Abstract]) OR (cafe[Title/Abstract]) OR (coffee[Title/Abstract])) AND ((cohort study[Title/Abstract]) OR (cohort study[MeSH Terms])) AND (("0001/01/01"[PDat] : "2019/09/30"[PDat]))

Supplementary Table 1. The detail demographic information of the study population from the included studies

| Study | Amount of Coffee | Participants | Male (%) | Age | HTN | DM | HL | BMI | Smoking (%) | Alcohol  (g/week) |
| --- | --- | --- | --- | --- | --- | --- | --- | --- | --- | --- |
| de Koning Gans | <1 | 7452 | 21 | 46.3±14.1 | 34.9 | 1.6 | 7.0 | - | 22.3 | 52.7 |
|  | 1-2 | 5023 | 19 | 49.5±12.5 | 37.2 | 1.7 | 6.7 | - | 23.1 | 73.9 |
|  | 2.1-3 | 5063 | 20.8 | 49.8±11.9 | 38.7 | 1.4 | 7.6 | - | 25.3 | 77.8 |
|  | 3.1-4 | 6477 | 19.7 | 51.7±11.0 | 39.4 | 1.4 | 6.7 | - | 25.1 | 75.3 |
|  | 4.1-6 | 8906 | 29.6 | 49.6±10.5 | 36.3 | 1.6 | 8.8 | - | 36.6 | 88.5 |
|  | >6 | 4593 | 44.2 | 47.1±9.9 | 33.4 | 1.1 | 9.9 | - | 53.7 | 99.7 |
|  |  |  |  |  |  |  |  |  |  |  |
| Greenberg | <0.5 | 292 | 42.5 | 75.6±0.3 | 38.7 | 8.2 | - | 25.7±0.3 | 12.7 | 27.4* |
|  | 0.5-2 | 349 | 39 | 76.0±0.3 | 37.8 | 10.1 | - | 26.1±0.3 | 10 | 23.8* |
|  | 2-4 | 631 | 39.1 | 75.0±0.2 | 38.5 | 8.4 | - | 26.2±0.2 | 13.5 | 28.8* |
|  | >4 | 441 | 50.3 | 73.7±0.3 | 29.3 | 5.9 | - | 25.7±0.2 | 23.8 | 31.5* |
|  |  |  |  |  |  |  |  |  |  |  |
| Mineharu-men | <1/wk | 11680 | 100 | 59.5 | 20.6 | 8.9 | - | 22.6 | 46.7 | 75.3* |
|  | 1-6/wk | 8837 | 100 | 57.4 | 21.3 | 7.0 | - | 22.8 | 51.3 | 78.7* |
|  | 1-2/day | 8012 | 100 | 55.5 | 16.6 | 6.0 | - | 22.7 | 58.9 | 76.1* |
|  | >3/day | 3061 | 100 | 51.7 | 10.7 | 5.5 | - | 22.6 | 76.8 | 67.4* |
|  |  |  |  |  |  |  |  |  |  |  |
| Mineharu-women | <1/wk | 18214 | 0 | 60.2 | 28.1 | 5.5 | - | 23 | 3.5 | 17.7* |
|  | 1-6/wk | 11966 | 0 | 56.9 | 22.0 | 3.2 | - | 23.1 | 3.8 | 26.9* |
|  | 1-2/day | 12477 | 0 | 55.3 | 18.3 | 3.2 | - | 22.8 | 6.5 | 30.5* |
|  | >3/day | 2732 | 0 | 50.9 | 12.1 | 2.5 | - | 22.7 | 18.3 | 34.3* |
|  |  |  |  |  |  |  |  |  |  |  |
| Lopez-Garcia | <1/m | 303293 | 0 | 55 | 17 | 2 | 26 | 25.7 | 8 | 2.8 |
|  | 1/m-4/wk | 236388 | 0 | 56 | 19 | 2 | 30 | 25.6 | 9 | 4.0 |
|  | 5-7/w | 586501 | 0 | 56 | 18 | 2 | 30 | 25.6 | 12 | 5.3 |
|  | 2-3/d | 541298 | 0 | 56 | 14 | 2 | 26 | 25.1 | 22 | 6.6 |
|  | >4/d | 222706 | 0 | 56 | 10 | 1 | 21 | 24.7 | 39 | 5.8 |
|  |  |  |  |  |  |  |  |  |  |  |
| Kobubo | 0 | 19841 | 42.2 | 56.6 | 25.2 | 7.1 | 5.9 | 23.5 | 16.9 | 37.7* |
|  | 1-2/wk | 18762 | 45.0 | 55.2 | 22.1 | 4.8 | 5.7 | 23.8 | 20.2 | 41.6* |
|  | 3-6/wl | 13364 | 51.7 | 53.5 | 17.0 | 4.1 | 4.4 | 23.7 | 26.7 | 47.5* |
|  |  |  |  |  |  |  |  |  |  |  |
| Flogel | <1/d | 8689 | 43.9 | 49.9 ± 8.6 | 37.6 | - | - | 25.8 | 13.2 | 13.5 |
|  | 1-2 | 3860 | 41.1 | 49.2 ± 8.5 | 38.5 | - | - | 25.8 | 11.7 | 15.3 |
|  | 2-3 | 10617 | 37.2 | 49.8 ± 8.7 | 38.2 | - | - | 25.8 | 12.0 | 15.9 |
|  | 3-4 | 7356 | 42.6 | 49.0 ±8.2 | 35.4 | - | - | 25.8 | 13.8 | 16.8 |
|  | >4 | 12137 | 43.1 | 50.2±8.4 | 34.9 | - | - | 26.2 | 15.7 | 16.0 |
|  |  |  |  |  |  |  |  |  |  |  |
| Larsson | <1 | 33807 py | 0 | 61.6 | 20.6 | 3.4 | - | 25.0 | 16.4 | 3.6 |
|  | 1-2 | 132857 | 0 | 62.2 | 21.3 | 3.3 | - | 24.9 | 17.9 | 4.6 |
|  | 3-4 | 135678 | 0 | 61.3 | 18.9 | 3.0 | - | 25.0 | 23.9 | 4.2 |
|  | >5 | 56671 | 0 | 59.2 | 18.0 | 3.1 | - | 25.2 | 37.4 | 3.8 |

HTN, hypertension; DM, diabetes mellitus; HL, hyperlipidemia; BMI, body mass index

*, >1 glass of alcoholic drink/d (%)
